# Advances in Nutrition, Dietary Supplements and Ergogenic Aids for Athletic Performance: Trends and Future Prospects

**DOI:** 10.3390/nu15102246

**Published:** 2023-05-09

**Authors:** Diego A. Bonilla, Daniel Boullosa, Juan Del Coso

**Affiliations:** 1 Research Division, Dynamical Business & Science Society—DBSS International SAS, Bogota 110311, Colombia; 2 Research Group in Biochemistry and Molecular Biology, Universidad Distrital Francisco José de Caldas, Bogotá 110311, Colombia; 3 Research Group in Physical Activity, Sports and Health Sciences (GICAFS), Universidad de Córdoba, Montería 230002, Colombia; 4 Faculty of Physical Activity and Sports Sciences, Universidad de León, 24004 León, Spain; daniel.boullosa@gmail.com; 5 Human Movement and Sports Performance Analysis (AMRED), Universidad de León, 24004 León, Spain; 6 Centre for Sport Studies, Rey Juan Carlos University, 28943 Fuenlabrada, Spain; juan.delcoso@urjc.es

Sports nutrition is a scientific discipline that explores the relationship between nutrients and physical exercise performance. It examines how nutrients such as carbohydrates, proteins, fats, vitamins, and minerals affect energy metabolism, muscle function, and recovery during exercise. Sports nutrition research aims to determine the optimal nutrient intake for athletes following their training, performance goals, and body composition. Additionally, it considers the impact of various dietary patterns, such as plant-based and ketogenic diets, on athletic performance. Overall, it seeks to understand how to optimize dietary intake to support athletic performance and maintain or enhance health status [1]. Recently, the prospect of using in sports nutrition to reduce the risk of injury and optimize recovery in athletes has increasingly been studied. For instance, ingesting a post-match recovery beverage containing native whey protein and carbohydrates may reduce exercise-induced elevation in muscle damage indicators and sustain physical performance in rugby players [2]. In injured athletes, a comprehensive systematic integrative review by Giraldo-Vallejo et al. (2023) showed the positive impact of energy availability, and high protein and carbohydrate diets content, as well as the potential use of collagen, creatine monohydrate, omega-3 (fish oils), and vitamin D to support tissue repair and reduce inflammation [3].

Incorporating dietary supplements into a sports nutrition plan can be beneficial for athletes looking to optimize their performance and health status, but it is also important to use supplements responsibly and under appropriate guidance. Available evidence suggests that nutritional supplements tailored to athletes’ needs can promote muscle growth, enhance athletic performance, and facilitate post-exercise recovery [4]. Systematic reviews and meta-analyses support the potential of creatine monohydrate for improving power- and strength-related variables [5,6], caffeine for endurance running events [7], beta-alanine for exercise capacity and performance [8], and dietary nitrate for repeated high-intensity activities such as combat sports [9]. Consuming high doses of caffeine may have the potential to improve muscular strength in resistance-trained participants [10]. This corroborates the findings of a recent meta-analysis by Delleli et al. (2023) showing that caffeine may be also ergogenic for certain power- and strength-related aspects of combat sports performance [11].

It is considered acceptable for the athletes with nutritional supervision to include the strategic use of a small number of nutritional supplements under a low-risk “Food First but Not Always Food Only” framework [12]. Practitioners and athletes should be cautious of the high percentage of commercialized dietary supplements that are only supported by limited scientific evidence and the potential risk of supplement contamination with substances prohibited by the World Anti-Doping Agency. In this regard, it has been shown that understanding of essential anti-doping regulations and strategies for preventing doping among Spanish sports science majors is inadequate, which suggests that there is a need to introduce specialized anti-doping training in the curriculum of undergraduate students studying sports sciences [13].

The field of sports nutrition is an evolving and dynamic area of research that draws upon various disciplines, such as exercise physiology, biochemistry, and human nutrition. This multidisciplinary approach has enabled researchers to deepen their understanding of the role of nutrients in sport to enhance athletic performance and recovery. There has been a consistent increase in the number of scientific publications on the topic of sports nutrition in recent years [14]. A bibliometric analysis on VOSviewer v1.6.19 (Centre for Science and Technology Studies, Leiden University, The Netherlands) indicates that this trend is continuing, with an increasing number of studies on new technologies, energy availability, para-athletes, societies, and adolescents published during the period 2022–2023 (see Figure 1). Sports nutrition encompasses a broad range of topics. However, the expanding body of literature in sports nutrition still underscores the significance of this field in optimizing the health and performance of athletes and the general population.

There are still limited studies on the impact of sports nutrition in various areas that can significantly affect an athlete’s nutrient requirements and performance, e.g., exercise in hot and humid environmental conditions, exercise at high altitudes, and high-intensity intermittent group exercise (e.g., dance exercise or fitness classes). Additionally, female, child, and adolescent athletes have specific nutrient requirements that require further research. Co-supplementation with different nutrients and the impact of sports nutrition on immune function, injury prevention, and mental health are also topics that require more research. Researchers and practitioners are encouraged to read the Strategic Planning for 2022–2026 of the Office of Dietary Supplements (ODS) at the National Institutes of Health (NIH) since the NIH Dietary Supplement Research Coordinating Committee (DSRCC) has been established to increase the information exchange, communication, collaboration, and coordination of dietary supplements and total dietary intake research/training activities [15]. Thus, ongoing research in sports nutrition is crucial for providing evidence-based and evidence-oriented recommendations to practitioners and optimizing nutrient intake strategies to support athletic performance, enhance recovery, and promote health and well-being.

## Figures and Tables

**Figure 1:**
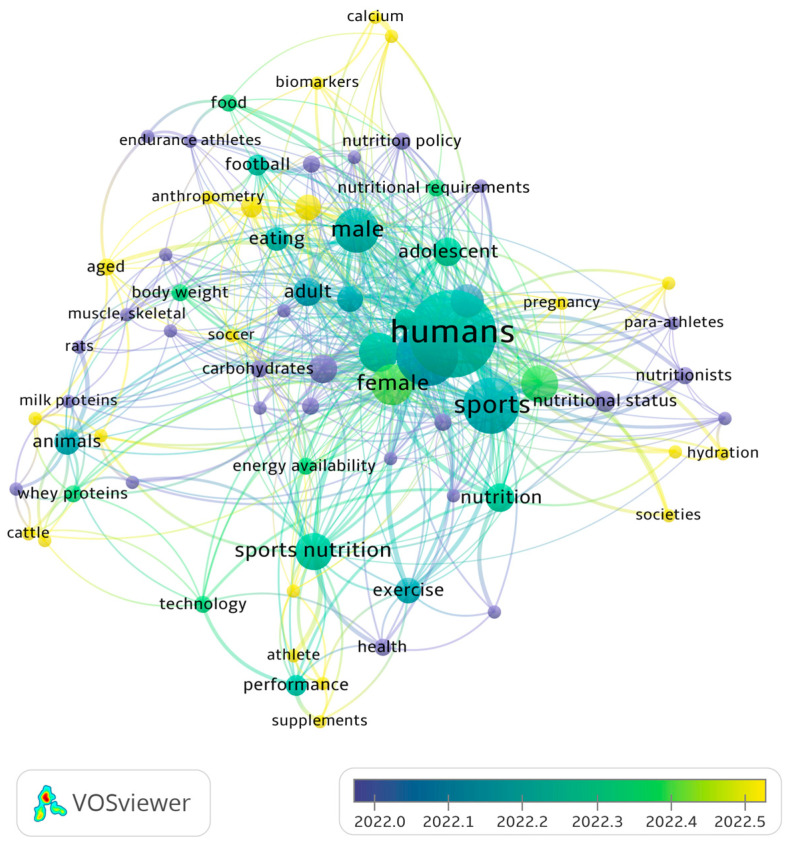
Bibliometric analysis of keyword co-occurrences in sports nutrition in recent years. The search was performed as “sports nutrition AND supplementation” using the API of the open access repository Europe PubMed Central. The size of nodes represents the weight based on occurrences. The connections between the nodes illustrate their co-occurrence in the same article (https://www.vosviewer.com/, accessed on 15 April 2023).
